# V-ATPase and Lysosomal Energy Sensing in Periodontitis and Medicine-Related Osteonecrosis of the Jaw

**DOI:** 10.3390/biom15070997

**Published:** 2025-07-11

**Authors:** Xianrui Yang, Lexie Shannon Holliday

**Affiliations:** Department of Orthodontics, University of Florida College of Dentistry, Gainesville, FL 32610, USA; xyang@dental.ufl.edu

**Keywords:** vacuolar H^+^-ATPase, AMPK, AMP-activated protein kinase complex, mTORC1, mammalian target of rapamycin complex 1, periodontal disease, regulator, Rheb, RAG GTPase, medicine-related osteonecrosis of the jaw

## Abstract

Diabetes is a risk factor for periodontitis. Increasing evidence suggests that a central player in this link is the vacuolar H+-ATPase (V-ATPase), which provides a physical and functional core for regulation by the catabolic lysosomal AMP-activated protein kinase complex (L-AMPK) and the anabolic mammalian target of rapamycin complex 1 (mTORC1). These complexes detect levels of various cellular nutrients, including glucose at the lysosome, and promote cellular responses to restore homeostasis. The high-glucose conditions of diabetes foster anabolic mTORC1 signaling that increases inflammation and inflammatory bone resorption in response to periodontal infections. Here, we review the structure and composition of V-ATPase, L-AMPK, mTORC1, and other elements of the energy-sensing platform. Mechanisms by which V-ATPase passes signals to the complexes are examined and recent data are reviewed. Current anti-bone resorptive therapeutics, bisphosphonates and denosumab, enhance the risk of medicine-related osteonecrosis of the jaw (MRONJ) and are not used to treat periodontal bone loss. Accumulating data suggest that it may be possible to target inflammatory bone resorption through agents that stimulate L-AMPK, including metformin and glucagon-like peptide-1 agonists. This approach may reduce inflammatory bone resorption without major effects on overall bone remodeling or increased risk of MRONJ.

## 1. Introduction

Irving Glickman, who is known as the “father of modern periodontology”, observed a heightened incidence of periodontitis in people suffering from diabetes [1,2,3]. We increasingly understand the underlying mechanisms for this connection. One component is cellular energy sensing at the lysosome [4]. During the past thirty-five years, a series of advances have led to the view that in addition to being an essential proton pump, vacuolar H^+^-ATPase (V-ATPase) is also vital for lysosomal energy sensing [4]. As a proton pump, V-ATPase is required for duties including acidifying lysosomes, late endosomes, phagosomes, autophagosomes, and compartments of the Golgi in eukaryotic cells [5]. In addition, V-ATPase pumps protons out of specialized cells in the kidney to help maintain systemic acid–base balance [6]. In osteoclasts, V-ATPase pumps protons into an extracellular resorption compartment that allows the removal of bone, the first step in bone remodeling [7]. In neurons, V-ATPase is crucial for generating an energy gradient that allows the loading of neurotransmitters into synaptic vesicles [8].

V-ATPases also play a central and ancient role in energy sensing [6,7,9]. Table 1 shows the percentage identity of selected components of the lysosomal energy-sensing apparatus in different eukaryotic organisms. Current understanding of these components and their interactions in mammals will be described in the article. V-ATPase interacts with two protein complexes, the catabolic lysosomal AMPK complex (L-AMPK) [10] and the anabolic mammalian target of rapamycin complex 1 (mTORC1) [11]. These interactions are in part through the Ragulator/Ras-related GTPase (RAG) complex, which is itself an amino acid sensor [12,13], and the scaffolding protein, axis inhibition protein (AXIN) [14]. The history of the discovery of mTORC1 and lysosomal AMPK and their characterization has recently been described authoritatively, and we will direct readers to these excellent review articles for detailed background [4,15,16]. In addition, V-ATPase and the Ragulator/RAG complex have also been recently excellently reviewed [5,6,17,18]. In this article, we will provide brief introductions to the central players, V-ATPase, mTORC1, L-AMPK, the Ragulator/RAG complex, and associated proteins. We will discuss evidence that lysosomal energy sensing is crucial in periodontal disease. We will focus on the interactions between the lysosomal energy-sensing platform components in the setting of periodontal disease and examine crucial open questions regarding the complexes and their functions.

## 2. Lysosomal Energy Sensing and Periodontitis

Sensing nutrients in cells occurs at multiple levels. Is it reasonable to hypothesize that energy sensing at the lysosome has an important or crucial role in periodontitis? The best evidence to date is that inhibitors that specifically or selectively affect lysosomal energy sensing have effects on the pathology of periodontal disease.

### 2.1. Rapamycin/Sirolimus

Rapamycin (also known as sirolimus) is a specific inhibitor of mTORC1, part of the lysosomal energy-sensing complex [19]. Various studies in animal models show that rapamycin ameliorates some of the pathological effects of periodontal disease, including bone loss [20,21,22]. This is likely due to reduced inflammatory signaling, decreased oxidative stress, and direct inhibition of the activity of osteoclasts [22]. A recent examination of the effects of off-label rapamycin use provides some evidence that it reduces periodontitis-linked bone loss in humans [23]. However, rapamycin has been reported to increase the risk of medicine-related osteonecrosis of the jaw (MRONJ), so caution is indicated in the use of this drug [24].

### 2.2. Metformin

Metformin less explicitly targets lysosomal energy sensing compared with rapamycin [25,26,27]. It affects the overall glucose supply and has direct effects on V-ATPases involved in energy sensing, as will be discussed in greater detail later in the article [28]. Various studies in animal models and a few human studies report that metformin is efficacious for the treatment of periodontitis [29,30,31,32,33,34,35,36,37,38]. Unlike rapamycin, there is no evidence that metformin triggers MRONJ, and indeed, some studies suggest that it may reduce the pathology of MRONJ [39,40,41]. The differences between metformin and rapamycin, which both result in a reduction in mTORC1 activity, may be due to metformin functioning to simultaneously increase AMPK activity while inhibiting mTORC1, serving to dial the natural rheostat toward catabolism, whereas rapamycin blocks one pathway without directly affecting other branches of the nutrient signaling network.

A challenge for the use of metformin or other L-AMPK modulators for periodontitis is that they affect all parts of the body. An exciting approach is to deliver metformin locally to the periodontium. Various approaches have been reported recently, including the use of synthetic scaffolds or the inclusion of metformin in organic matrices [31,42,43,44,45,46,47,48,49,50]. Another potential approach is based on the finding that metformin stimulates certain cell types to produce extracellular vesicles or cytokines that reduce the pathology of periodontitis [33,36]. These extracellular vesicles or cytokines could be used to treat periodontitis. Recent studies have examined the mechanisms by which metformin ameliorates periodontitis [33,42,50,51,52,53,54,55,56,57,58].

### 2.3. Glucagon-like Peptide-1 (GLP-1) Agonists

GLP-1 agonists, including Ozempic, Wegovy, Rybelsus, Saxenda, Victoza, Trulicity, Byetta, and Bydureon, have stormed the market [59,60]. Since the introduction of the first of this class, Byetta [61,62], in 2005, GLP-1 agonists have become mega drugs. Although these therapeutics are approved for treating diabetes, they have become popular for off-label use to reduce obesity. The world market in 2024 was USD 53.5 billion and is projected to rise to USD 156 billion by 2030 [63]. These drugs work by increasing insulin secretion and reducing glucagon [59]. This reduces blood glucose. Consequently, they stimulate L-AMPK and inhibit mTORC1 [64]. They are not thought of as agents to treat periodontal disease or MRONJ but would be expected to do so if lysosomal energy sensing played an important role. Several studies now show that they do reduce periodontal infections and associated pathology [65,66,67]. There is also some evidence that periodontal infection alters regulation through the GLP-1 receptor [68,69,70,71,72]. There is no evidence to date regarding the potential effects of GLP-1 agonists on MRONJ.

### 2.4. Salicylate

Salicylate is the active compound in aspirin. In addition to its well-known role as a cyclooxygenase inhibitor, salicylate is a direct stimulator of AMPK [73]. Evidence suggests it may be beneficial for the treatment of periodontal disease [74,75]. Efforts are underway to develop methods to apply salicylate directly to the periodontium as an approach to treating periodontal disease [74,76]. To date, salicylate has not been implicated in the development of MRONJ.

### 2.5. Resveratrol

This small molecule, which is famously present in red wine, stimulates Sirtuin-1, a deacetylase that promotes anti-inflammatory pathways [77,78,79,80,81]. Two mechanisms that have been described are likely to impact periodontal bone loss. Resveratrol stimulates Sirtuin-1 to deacetylate Forkhead Box Os (FOXOs), transcription factors involved in processes including energy metabolism [82]. Deacetylation of FOXOs by this route has been described to inhibit osteoclastic bone resorption [82]. As will be described in detail below, Sirtuins were recently shown to inactivate V-ATPase activity by deacetylating several residues on the E-subunit of V-ATPase. This promotes L-AMPK activity [83]. This would also be expected to directly inhibit V-ATPase activity in osteoclasts, which is required for bone resorption [84]. Resveratrol has been described to have therapeutic effects on periodontal disease [81,85,86,87,88]. Of particular importance, it has recently been shown that genetic variants of the Sirtuin-1 gene increase the risk of the development of MRONJ [89,90]. Menaquinone prevented MRONJ in a mouse model by a Sirtuin-dependent mechanism [91].

### 2.6. 2-Deoxy-D-Glucose

Data were presented supporting the glycolysis inhibitor 2-Deoxy-d-glucose (2-DG) as a therapeutic agent to reduce the pathological effects of periodontal disease [92,93]. 2-DG enters cells through the same transporters as glucose but does not enter glycolysis and accumulates in cells, reducing glycolytic production of ATP and stimulating AMPK [94]. While this requires being used carefully, as it can also trigger apoptosis and is not cell-type-specific, it provides additional evidence linking glycolytic metabolism and therapeutic effects that are useful for treating periodontitis.

### 2.7. Dorsomorphin (Compound C)

There has been less interest in developing inhibitors of AMPK or stimulators of mTOR. The data that is available is consistent with the overall premise that, in periodontal disease, stimulating catabolic pathways is the best target for reducing the pathology associated with periodontal infections. Perhaps the most obvious of these results is Dr. Glickman’s observation that diabetics have an increased risk of periodontal disease [1]. Dorsomorphin is an inhibitor of AMPK. It has been shown to reduce osteoclast differentiation and activity in vitro [95]. It has not been tested on periodontal disease, but would be expected to exacerbate the pathology.

## 3. Key Players in Lysosomal Energy Sensing

Energy sensing in general and lysosomal energy sensing in particular is extraordinarily complex. The primary components are described below. These include V-ATPase, which serves as the central element of the lysosomal energy-sensing machinery, mTORC1, which interacts with V-ATPase when energy is abundant, and L-AMPK, which interacts with a different activation state of V-ATPase when energy is depleted. We will also discuss the Ragulator/RAG complex, which regulates the activation state of mTORC1, and what is known about how mTORC1 and L-AMPK interact with V-ATPase. We will discuss how the glycolytic enzyme aldolase binds V-ATPase. Finally, we will address what is known about the reversible assembly of V-ATPase and how this may provide crucial signals for determining the balance between mTORC1 and L-AMPK activity.

### 3.1. V-ATPase

To understand lysosomal energy sensing, it is crucial to be familiar with V-ATPase. It interacts with both mTORC1 and L-AMPK, and its activation state and (probably) its assembly state are linked to mTORC1 and L-AMPK activity. We will review the overall structure of V-ATPase in this section.

**Figure 1:**
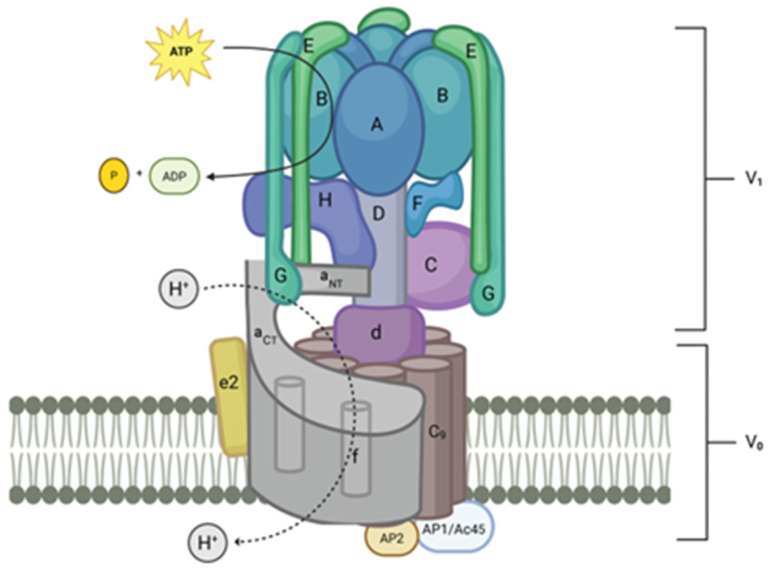
Model of mammalian V-ATPase. The V1 component is in the cytosol of the cell. V0 is composed of membrane-spanning elements. Rotary movement of the D-subunit and d-subunit powered by ATP hydrolysis in the A-subunit causes rotation of the c-subunit, c″-subunit ring. This rotating ring forms with the a-subunit, a rotation-dependent channel for protons to move through the membrane. Protons are pumped into lumen of vesicles or organelles. In the case of specialized cells, including osteoclasts and renal intercalated cells, protons are pumped out of the cell through the plasma membrane (created with BioRender.com).

Mammalian V-ATPases are composed of 15 subunits divided into a peripheral domain called V1 and an integral domain called V0 (Figure 1) [5,6]. As shown in Table 2, many of the subunits are present as isoforms that include one that is ubiquitous and one or more isoforms that are found in specialized V-ATPases in specific cell types. V1 contains eight proteins with the following stoichiometry: three A-subunits, three B-subunits, one C-subunit, one D-subunit, three E-subunits, one F-subunit, three G-subunits, and one H-subunit.

The structure of V-ATPases from budding yeast and mammalian brains was recently determined by cryo-electron microscopy and X-ray crystallography, providing excellent models and new insight into these enzymes [8,96,97]. The A-subunits, the site of ATP hydrolysis, are organized into a hetero-hexagon with three B-subunits. Three extended dimers of the E-subunit and G-subunit attach to the top of each B-subunit designated EG1, EG2, and EG3 based on their differential association with the membrane proximal collar domain, which is composed of an a-subunit, C-subunit and H-subunit. EG1 binds the H-subunit and a-subunit. EG2 binds the C-subunit foot domain and a-subunit and EG3 binds the C-subunit head domain and a-subunit [5]. The C-subunit and the H-subunit form a platform at the membrane side of the A/B hetero-hexagon and interact with both V1 and V0. The D-subunit forms a rotor that transduces conformational changes in the hetero-hexagon to spin; it is linked to the V0 through the d-subunit. The V0 domain includes the largest subunit, the a-subunit, which has a membrane-spanning region that contains nine transmembrane domains and a cytosolic region that forms a base interacting directly with various subunits, including the E-subunit/G-subunit stators. It functions in conjunction with a ring of nine c-subunits plus one c″-subunit (the gen is called *ATP6V0b*) in mammals, which is spun by the D-subunit/d-subunit rotor.

**Table 2:** V-ATPase subunits, their isoforms, isoform expression, and diseases caused by mutations in the genes.

Gene	Protein	Location	Disease
*ATP6V1A*	A	ubiquitous	Developmental and epileptic encephalopathies, cutis laxa, cardiac abnormalities, dysmorphic facial features, and severe hypotonia lysosome dysfunction [98,99]
*ATP6V1B1*	B1	kidney, epididymis	Renal tubular acidosis with deafness [100]
*ATP6V1B2*	B2	ubiquitous	Dominant Deafness–Onychodystrophy Syndrome (DDOD), Zimmermann–Laband Syndrome 2 (ZLS2), DOORS (Deafness, Onychodystrophy, Osteodystrophy, Intellectual Disability, and Seizures [101,102,103]
*ATP6V1C1*	C1	ubiquitous	DOORS syndrome (Deafness, Onychodystrophy, Osteodystrophy, Impaired Intellectual Development, And Seizures Syndrome), autosomal recessive osteopetrosis, and other lysosomal storage disorders [102]
*ATP6V1C2*	C2	lung, kidney	Renal tubular acidosis with deafness [104]
*ATP6V1D*	D	ubiquitous	
*ATP6V1E1*	E1	testis	Autosomal recessive cutis laxa type Iic [103]
*ATP6V1E2*	E2	ubiquitous	
*ATP6V1F*	F	ubiquitous	
*ATP6V1G1*	G1	ubiquitous	
*ATP6V1G2*	G2	neural	
*ATP6V1G3*	G3	kidney, epididymis	
*ATP6V!H*	H	ubiquitous	lysosome dysfunction [98,99]
*ATP6V0a1*	a1	ubiquitous	Developmental and epileptic encephalopathy: early-onset seizures, developmental delays, intellectual disabilities, neurodegenerative disorders bone, kidney disorders. Neurodegenerative disorders: Parkinson’s disease, Alzheimer’s disease, and other conditions affecting the brain [105,106]
*ATP6V0a2*	a2	ubiquitous	Autosomal recessive cutis laxa type IIA (ARCL2A) and, in some cases, wrinkly skin syndrome [107]
*ATP6V0a3*	a3	osteoclast (ubiquitous?)	Autosomal recessive osteopetrosis [108]
*ATP6V0a4*	a4	kidney, epididymis	Autosomal recessive distal renal tubular acidosis, renal tubular acidosis with deafness [109]
*ATP6V0b*	b, c″	ubiquitous	
*ATP6V0c*	c	ubiquitous	
*ATP6V0d1*	d1	ubiquitous	
*ATP6V0d2*	d2	kidney, epididymis	
*ATP6V0e*	e	ubiquitous	
*ATP6AP1*	AP1, AC45	ubiquitous	Congenital Disorder of Glycosylation type, Follicular lymphoma, immunodeficiency with hepatopathy, cognitive impairment [110,111,112]
*ATP6AP2*	AP2, Prorenin Receptor	ubiquitous	X-linked syndromic intellectual disability (Hedera type), X-linked Parkinsonism–spasticity syndrome, and congenital disorder of glycosylation type 2R autophagic liver disease [113,114]

With the a-subunit’s transmembrane domain, this spinning creates a route for protons through the membrane [115]. V0 also contains an e-subunit that is located in association with the a-subunit. In addition, two “accessory” proteins, AC45 (*ATP6AP1*) and the pro-renin receptor (*ATP6AP2*), were recently identified as having transmembrane domains that are within the c-subunit ring in intact bovine brain V-ATPases and seem to be true mammalian V-ATPase subunits [8,97]. As we will see, *ATP6AP1* plays a crucial role in energy sensing in the activation of mTORC1 [116,117].

Because V-ATPases have multiple isoforms of numerous subunits, and many cell types express multiple isoforms, the question is whether those V-ATPases that interact with energy-sensing complexes contain specific isoforms that are involved in their interaction with the energy-sensing machinery. This is not yet clear. One candidate for a lysosomal sensing subunit is the a3-isoform of the a-subunit [118,119]. The a3-subunit is overexpressed in osteoclasts, where it is part of the subset of V-ATPases that are targeted to the ruffled border subdomain of the plasma membrane, and it is required for bone resorption [120,121]. The a3-subunit is often described as the lysosomal isoform of the a-subunit [118]. Null mutations in the gene lead to a very severe form of osteopetrosis, but children with the mutation often survive several years and die from complications of the bone defects that result from inactive osteoclasts [120,121]. Bone marrow transplants can extend life for extended periods [122,123]. Five-year survival with a 100% compatible donor is reported to be 73% [122,123,124]. No metabolic syndrome pathology has been reported.

The hypothesis that interactions between mTORC1 and L-AMPK might be mediated by specific isoforms of V-ATPase subunits is supported by a recent study that shows that TBC/LysM-Associated Domain Containing 2 (TDLc2) binds selectively to the B1-isoform of the B-subunit, which is expressed in the kidney. TDLc2 binds B1 and inactivates the V-ATPase through the interaction [125]. Knockout of TDLc2 in mouse kidneys impairs urine alkalinization [126].

Mammalian Enhancer-of-Akt-1–7 (mEAK7) binds the A-, B-, E- and D-subunits [127]. It may serve as a V-ATPase activator and as a non-canonical means of linking mTOR signaling to V-ATPase activity [125]. In Section 3.7, we will discuss reversible assembly of V-ATPase as a regulatory mechanism and how it may be linked to the formation of the mTORC1/V-ATPase and L-AMPK/V-ATPase complexes.

### 3.2. mTORC1

The mTORC1 complex stimulates anabolic signaling when nutrients are plentiful. The central component is the enzyme mTOR. Active mTORC1 complexes form and are maintained in association with active V-ATPase in the lysosome. In this section, we will discuss how mTORC1 fits into mTOR signaling in general and describe the composition of the mTORC1 complex. We will use the second mTOR complex, mTORC2, for comparison.

mTOR was discovered through studies of the activity of rapamycin, a bacterial toxin discovered on Easter Island (Rapa Nui is its native name), which was first described as having strong anti-fungal properties [128]. It was later shown to also have anti-cancer and anti-inflammatory activities [129,130]. This led to the concept and demonstration that rapamycin had the same target in yeast and mammals and the discovery of the conserved kinase, mTOR [129,131,132,133]. Subsequent studies have shown that rapamycin targets only one of two mTOR-containing complexes in mammals, mTORC1, which is composed of the catalytic mTOR subunit, regulatory-associated protein of mTOR (RAPTOR), mammalian lethal with sec-13 protein 8 (MLST8), proline-rich Akt substrate of 40 kDa (PRAS40), Tel2 interacting protein 1 (TTI1)/ Telomere maintenance 2 interacting protein 2 (TEL2) and DEP domain containing mTOR-interacting protein (DEPTOR) [134]. mTORC1 binds to the cytosolic region of V-ATPase through the Ragulator/RAG complex and AXIN in an amino-acid-dependent manner [11].

Active mTORC1 stimulates downstream signaling pathways that favor cell growth and proliferation. For example, many cancer cells display inappropriate mTORC1 activation under metabolic conditions where the catabolic L-AMPK signaling pathway should be favored [135,136,137]. mTORC1 is primarily involved in regulating protein synthesis, cell growth, and metabolism, and is activated by nutrients and growth factors [11].

mTORC2 is a second mTOR-containing complex that is composed of seven protein subunits [138,139]. These include the catalytic mTOR subunit, DEPTOR, mLST8, and TTI1/TEL2 complex, which are shared by both mTORC2 and mTORC1. Rapamycin-insensitive companion of mTOR (RICTOR), mammalian stress-activated protein kinase interacting protein 1 (mSIN1), and protein observed with Rictor 1 and 2 (Protor1/2) can only be found in mTORC2. As indicated by its name, RICTOR serves to protect mTOR in mTORC2 from rapamycin [140]. mTORC2 plays a more prominent role in cell survival and proliferation and is activated by stress and growth factors [139].

### 3.3. L-AMPK

The L-AMPK complex has at its core the enzyme AMPK. In this section, we will discuss the composition of L-AMPK and discuss how it can be activated, comparing it with the canonical AMPK stimulation pathway [4,141].

AMPK is a heterotrimeric protein complex formed by the catalytic α-subunit and regulatory β- and γ-subunits [10]. AMPK stimulates several signaling pathways that lead to catabolic responses. Canonical AMPK activation is triggered by an increase in the AMP/ATP and ADP/ATP ratios, which cause the binding of AMP to the AMPK γ-subunit. This activates the kinase through phosphorylation by liver kinase B1 (LKB1). It is now known that there are several non-canonical routes for AMPK activation that are linked to a V-ATPase-associated L-AMPK complex [142].

L-AMPK activation pathways were only recently elucidated [28,142]. When energy levels are low, myristoylated AMPK associated with lysosomes binds V-ATPase in a complex that includes aldolase, the Ragulator, AXIN, and the kinase, LKB1 [4,9,10,142,143]. Upon glucose starvation, AMPK is activated by phosphorylation on threonine 172, and its kinase activity triggers signaling pathways that promote catabolism and inhibit anabolic processes. Low-dose metformin primarily stimulates the L-AMPK pathway [28]. This occurs through the interaction between metformin and a protein called presenilin enhancer 2 (PEN2), which in turn interacts with AC45 to inhibit V-ATPase activity [28], activating L-AMPK. Important consequences of low-dose metformin include reducing glucose production in the liver, increasing insulin sensitivity in various body tissues, and reducing Growth Differentiation Factor 15 (GDF15) secretion, which reduces appetite [28].

**Figure 2:**
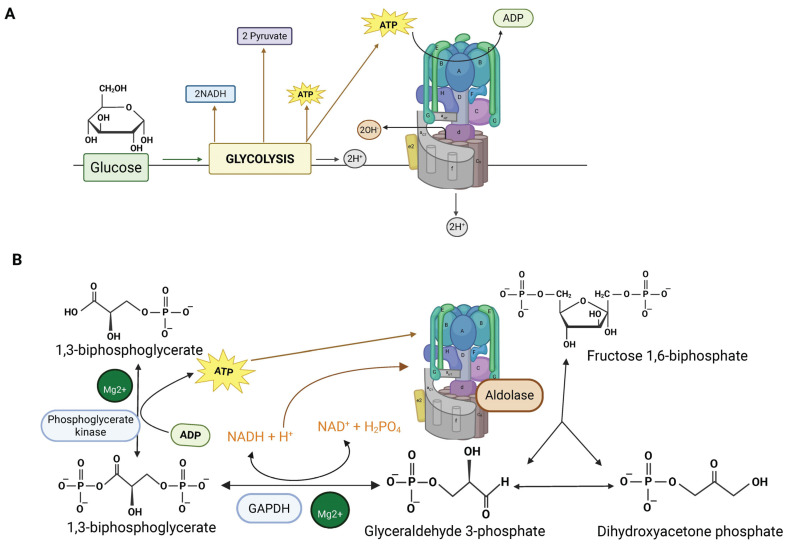
(**A**) Glycolysis produces both ATP to power the proton pump and protons. Local production of both protons and ATP by glycolytic enzymes linked to V-ATPase may reduce cytosolic alkalinization. This figure was adapted from reference [114]. (**B**) Focus on steps 5–7 of glycolysis which are proposed to be crucial for coupling glycolysis to V-ATPase. Aldolase, which is bound to V-ATPase, produces glyceraldehyde 3-phosphate and dihydroxyacetone phosphate (which interconvert). Glyceraldehyde 3-phosphate is converted to 1,3-biphosphate in step 6 by GAPDH, producing a free proton from phosphate. Step 7, which is coupled to step 6, produces 3-phosphoglycerate and an ATP. The step-6 and -7 reactions occur twice, giving a total of two ATPs and two free protons (created with BioRender.com).

Fructose-bisphosphate aldolase (Aldolase) was recently identified as an important component of L-AMPK signaling. The binding between Aldolase and the V-ATPase was first identified in the early 2000s [144]. The interaction was initially detected in yeast two-hybrid assays, then confirmed by various methods, including immunoprecipitations from osteoclasts. It was suggested that coupling glycolysis directly to the V-ATPases might be a means for preventing alkalinization of the cytosol [144]. Because glycolysis produces protons as a byproduct of ATP synthesis, having both ATP and protons produced in the microenvironment of V-ATPase might be very useful (Figure 2A) [144]. It is important to consider the steps immediately after step 5 of glycolysis, which is catalyzed by aldolase. Step 6 is catalyzed by glyceraldehyde 3-phosphate dehydrogenase (GAPDH), and phosphoglycerate kinase catalyzes step 7. Steps 6 and 7 are coupled reactions and produce a free proton, derived from inorganic phosphate. The phosphate is attached to glyceraldehyde 3-phosphate to give 1,3-bisphosphoglycerate. In step 7, an ATP produced as a phosphate from 1,3-bisphosphoglycerate is added to ADP (Figure 2B). Step 6 has a free energy of +6.3 KJ/mol, while step 7 has a favorable free energy of −18.8 KJ/mol. Coupling the two reactions allows them to proceed and links proton and ATP production. Finally, it was reported that GAPDH is bound to V-ATPase in addition to aldolase, further supporting the coupling of glycolysis to V-ATPase [145].

Carbonic anhydrase plays a role in maintaining cytosolic pH by converting CO_2_ produced by mitochondrial metabolism, plus water, to bicarbonate and protons. Mutations in carbonic anhydrase II result in distal renal tubular acidosis and mild osteopetrosis [112,146]. However, osteoclasts still function, although less efficiently, without carbonic anhydrase II, suggesting that other proton-generating strategies are available. Consistent with a role for localized glycolysis, Aldolase B was localized to the ruffled border of osteoclasts, the region rich in V-ATPases where protons are pumped into an extracellular resorption compartment during bone resorption [144].

**Figure 3:**
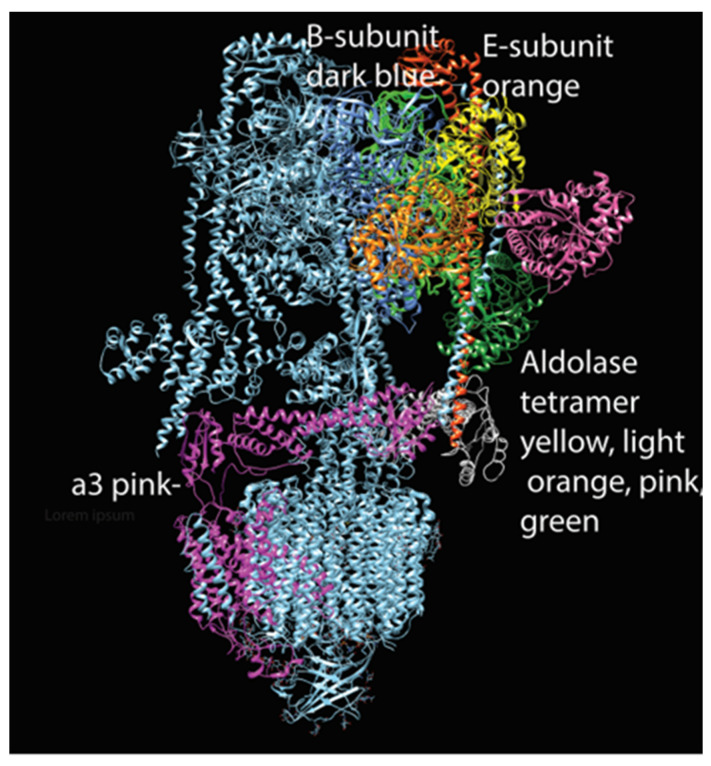
Model of aldolase tetramer attached to V-ATPase (created with Chimera). This hypothetical model suggests that an aldolase tetramer may be able to interact simultaneously with the three subunits shown to bind in in vitro studies [114].

Subsequent studies showed that aldolase is required in yeast for V-ATPase assembly, suggesting that the link between aldolase and V-ATPase, and indeed the link between V-ATPase and glycolysis, has ancient evolutionary origins [145,147]. Coupling V-ATPase to glycolysis may be a crucial means to prevent alkalinization of the cytosol whenever V-ATPase is active. In Figure 3, we provide a plausible binding interaction between V-ATPase and aldolase. This model is based on previous findings showing aldolase binding interactions with three subunits, the B-subunit and E-subunits of the V1 a-subunit [145].

Activated AMPK inhibits mTORC1 primarily by phosphorylating and activating the mTORC1-negative regulator TSC2 and by phosphorylating and inhibiting the mTORC1 subunit RAPTOR [148]. Activated L-AMPK promotes catabolic pathways, including pathways that break down molecules to produce ATP, such as fatty acid oxidation and glycolysis. L-AMPK stimulates autophagy and mitophagy to cannibalize cell components to provide basic nutrients. AMPK turns off pathways that require energy to build products, including gluconeogenesis (glucose production), lipogenesis (fat synthesis), and protein synthesis [4,142,149,150].

### 3.4. Ragulator/RAG Complex

The Ragulator/RAG complex serves to activate mTORC1 and to link mTORC1 to V-ATPase both physically and functionally. The Ragulator complex recruits the RAGs, small GTPases that in turn interact with another small GTPase, Ras homolog enriched in brain (Rheb). These receive signals from an amino acid transporter and the V-ATPase.

**Figure 4:**
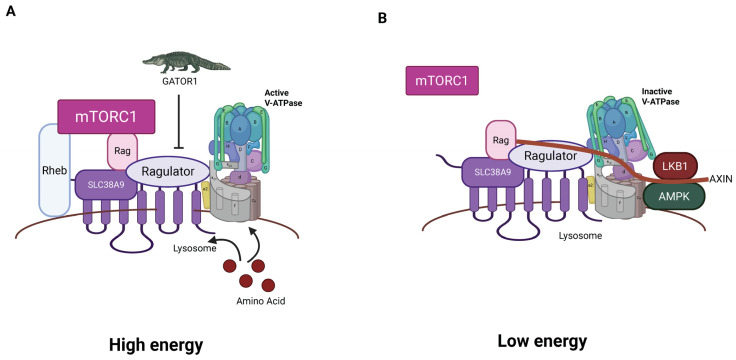
Model of mTORC1/Ragulator/RAG/L-AMPK complex. (**A**) In the high-energy condition in the presence of high sugars and amino acids, RAGS and Rheb are active and facilitate recruitment and activation of mTOR, driving anabolic pathways. The GAP GATOR1 acts as an inhibitor. (**B**) In the low-energy condition, the Ragulator is inactive, mTor is inactive, and the L-AMPK is recruited to V-ATPase through AXIN and activated by LKB1. The active AMPK stimulates catabolic pathways (created with BioRender.com).

The Ragulator is composed of five Late endosomal/lysosomal adaptor and MAPK and mTOR activator (LAMTOR) subunits [12,151]. A crystal structure shows that LAMTOR 2 and LAMTOR 3 form an obligate heterodimer and LAMTOR 4 and LAMTOR 5 form a second obligate heterodimer [152]. These two heterodimers interact. LAMTOR1 also forms a dimer with a different structure, which wraps the two heterodimers, stabilizing the structure. The Ragulator then recruits Ras-related-GTP binding (RAG) GTPases (Figure 4) [153].

The RAG GTPases represent a particularly complicated variation in the theme of regulation by the Ras family of small GTPases. Like other members of the Ras family, RAG GTPases have two states, GTP-bound or GDP-bound [154]. Transitioning occurs through guanine exchange factors (GEFs) that promote the exchange of the nucleotide. Because most free guanine nucleotides in the cytosol are GTP, GEFs promote activation, but as we will see below, there is a crucial variation on this theme. RAG GTPases, like other members of the family, require interaction with GTPase-activating proteins (GAPs) to convert GTP to GDP to change the activation state. RAG GTPases add additional levels of complexity to this basic scheme [153].

**Figure 5:**
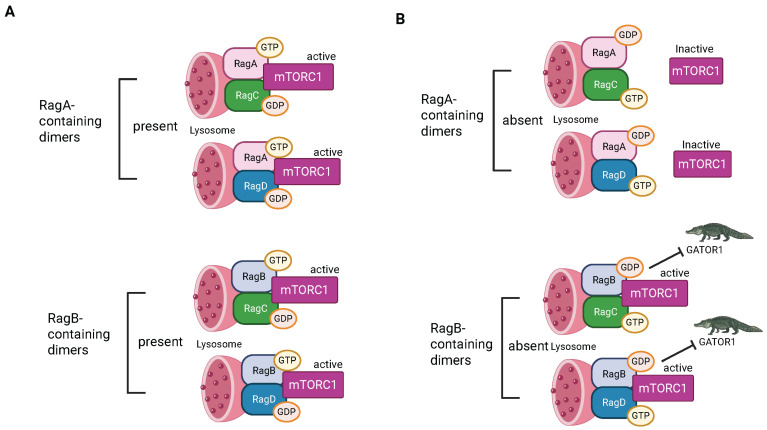
Regulation by RAG GTPases. (**A**) Instances where small RAGs are GTP-bound. (**B**) Instances when small RAGs are GDP-bound (created with BioRender.com).

There are four RAG GTPases: the small RAGs, RAGA and RAGB, and the large RAGS, RAGC and RAGD [151]. Small RAGs form heterodimers with large RAGs. Small RAGs are activated by the Ragulator, which serves as a GEF for RAGA/B but only triggers release of GTP from RAGC/D. Heterodimers in which the small RAG is GTP-bound and the large RAG is GDP-bound are active. When the large RAG is in its GTP-bound conformation, that heterodimer is locked into that state. Therefore, interaction with the Ragulator activates RAG GTPases by promoting GDP to GTP transition in the small RAGs by the typical strategy but triggers loss of GTP, not GDP, in the large RAGs [153] (Figure 5). Different RAG heterodimers have different activities; this regulation was the focus of a recent excellent review [17].

RAG heterodimers can also be activated by Solute carrier family 38 member 9 (SLC38A9) [153] (Figure 4). It binds and acts as a GEF for the small RAGs in the presence of the amino acid arginine. When the small RAG binds GTP, it breaks its interaction with SLC38A9, allowing SLC38A9 to activate more RAGs, acting like an enzyme. In the absence of amino acids arginine and leucine, GTPase-activating protein toward Rags 2 (GATOR2) promotes GTPase activity in the small RAGS, leading to inactivation [151]. This provides a pathway to inhibit Ragulator/RAG signaling and consequent activation of mTORC1 (as well as other anabolic signaling) when amino acids are limited.

The Ragulator with activated RAG heterodimer recruits mTORC1 to the lysosome [151]. It also recruits Rheb, another small GTPase, which allosterically activates the mTORC1 complex [155]. Activated mTOR in mTORC1 then activates pathways that result in anabolic processes [11]. These include protein synthesis by activating downstream effectors like S6K and 4E-BP, which promote translation and protein production [156]. Lipid synthesis is stimulated through SREBP transcription factors [134]. mTORC1 also stimulates nucleotide synthesis and mitochondrial biogenesis through transcriptional and translational regulation [157].

Like other members of the Ras family, a GEF is required to activate Rheb [100]. Recent data, surprisingly, identified the (or a) GEF as AC45, the newly identified subunit of the V-ATPase [116]. This may be related to the finding that concurrent mutations in RAGC and *ATP6AP1* occur in follicular lymphoma [158]. These make mTORC1 resistant to amino acid deprivation. The finding that AC45 is a GEF for Rheb will be discussed in more detail in the next section. The GAP for Rheb is the Tuberous Sclerosis Complex (TSC) 2-subunit of the TSC [159]. The TSC’s GAP activity is negatively regulated by insulin and other growth factors through PI 3-kinase/AKT signaling, keeping mTORC1 signaling on [160]. The GAP activity is positively regulated by LKB1/AMPK signaling, turning off mTORC1 signaling [148].

### 3.5. Associations Between V-ATPase, Ragulator, L-AMPK, and mTORC1

There remains considerable uncertainty regarding the binding interactions between components of the lysosomal energy-sensing apparatus. In this section, we will describe what is known and indicate areas that are unclear. The known structure of the V-ATPase serves as a guide to where interactions may occur.

There is an intrinsic asymmetry in the structure of V-ATPases [8,97]. The V-ATPase is mobile when V-ATPase is active, swaying gently as the stator arms resist the force generated by conformational changes associated with ATP hydrolysis and turning of the internal rotor [96,161]. This means that in an active pump, proteins that will be subjected to binding sites that may change rapidly in the active pump.

The three stator arms, composed of E- and G-subunits, connect the catalytic V1 domain to the membrane-embedded V0 domain [8,97]. As described in a previous section, the three stators have a unique membrane proximal attachment: EG1 binds the H-subunit and a-subunit, EG2 binds the C-subunit foot and a-subunit, and EG3 binds the C-subunit head and the N-terminus of the a-subunit [8,97]. The three attachment sites have the potential to be differentially regulated.

The V0 is dramatically asymmetrical. The most prominent element is the largest subunit, the a-subunit, which has an extensive membrane-embedded region which functions with the rotating ring of nine c-subunits with a single c″ in mammals to form the rotating membrane-embedded ring [8,97].

Current data suggest that mTORC1 interacts indirectly with V-ATPase through Ragulator/RAG, and that Ragulator interacts with AXIN, a scaffolding protein that also binds the B-subunit and C-subunit of V-ATPase and microfilaments [162], through binding between LAMTOR1 and AXIN [162]. As described above, AC45 was recently described as serving as a GEF for Rheb [117]. Rheb is recruited to the lysosome by active RAG heterodimers, which in turn are activated by interaction with the Ragulator complex and SLC38A9 [153].

The version of AC45 found in brain V-ATPases lacked its extracellular/luminal domain, which had been cleaved, leaving its C-terminal tail associated with V-ATPase. This included a transmembrane domain, with a short cytosolic element embedded in the c-ring of the V0 [8,97]. The pro-renin receptor also lacked its extracellular/luminal domain. The cleavage of the pro-renin receptor is through the action of furin and/or other proteases early in the secretory pathway, and this cleavage is crucial for V-ATPase function [163]. The short cytosolic domain of AC45 stimulates Rheb. The atomic level structure of the V0 component of brain V-ATPase shows AC45 off-center in the ring opposite the a-subunit. It is homologous with that of the yeast V-ATPase subunit Voa1 [164]. A challenge with the hypothesis that AC45 serves as a GEF for Rheb is that this region seems blocked by its location in the V-ATPase. The experiment in which AC45 was identified as a GEF for Rheb used overexpressed proteins [116]; it is possible that the interaction detected is not physiologically relevant. Alternatively, it may be exposed by a disassembled state of the V-ATPase. In any event, the activated Rheb then binds mTORC1 to activate the mTOR activity.

There is evidence that regulatory interactions between V-ATPase and mTORC1 may involve both activation of mTORC1 through V-ATPase activity and assembly of V-ATPase triggered by mTORC1 and associated proteins [9,165]. This will be examined in greater detail in the next section.

Recently it was reported that metformin acts directly on V-ATPase through an interaction with PEN2, a subunit of the γ-secretase [28]. This directly inhibits V-ATPase by binding AC45 and leads to activation of AMPK. This also might block the ability of AC45 to act as a GEF for Rheb, resulting in inhibition of mTORC1. Given the location of AC45 in V-ATPase, it is also possible that the association of PEN2 with AC45 triggered by metformin could disrupt the V0 domain of V-ATPase, inactivating the V-ATPase.

The γ-secretase is a multi-subunit transmembrane protease that cleaves the transmembrane domain of proteins [166]. Metformin had been shown to slightly increase the activity of γ-secretase, which is consistent with an interaction between the two [167]. Because γ-secretase activity contributes to the deposition of amyloid-β protein, this raised concerns regarding long-term use of metformin [167].

L-AMPK phosphorylates the A-subunit of V-ATPase [168]. This implies proximity to V-ATPase. It has been proposed that L-AMPK binds indirectly to V-ATPase through AXIN, which binds directly to V-ATPase and interacts with both L-AMPK and the Ragulator. AXIN binds the AMPK and LKB1 elements of the L-AMPK and serves to recruit them to V-ATPase in the lysosome in low-glucose conditions and to facilitate phosphorylation of AMPK by LKB1 in conjunction with inactivation of V-ATPase [14,146]. It must be noted that knockout of one of the two AXIN proteins, AXIN1, surprisingly, did not affect AMPK/mTORC1 signaling [169]. Separately, AXIN also has a role as an inhibitor of Wnt/β-catenin signaling, which is crucial for regulating osteoblast activity [14].

Both the C-subunit and the B-subunit of V-ATPase have been shown to bind microfilaments [170,171,172,173,174,175]. The binding site in the B-subunit is blocked in active V-ATPases by the stator arms containing an E-subunit and G-subunit, which contact the same region [8,96,97]. The C-subunit has been described as binding and crosslinking microfilaments in its disassembled state. It is possible that disassembly of V-ATPase leads to binding of microfilaments to B- and C-subunits and binding of AXIN to the partially disassembled V-ATPase, and then recruitment of AMPK and LKB1 to activate AMPK activity. The models that have been proposed rely on AXIN to serve as a scaffold for both the connection of L-AMPK and mTORC1, through the Ragulator [162]. AXIN was not detected associated with brain V-ATPases [8,97]. AXIN may only bind lysosomal V-ATPases, perhaps through a lysosome-specific isoform of V-ATPase.

Aldolase, as described above, is a key element of L-AMPK and was shown to bind three V-ATPase subunits: the a-subunit, the amino terminus of the B-subunit and the E-subunit [145]. For aldolase to utilize these three binding sites would suggest that binding is in the expected tetrameric form of active aldolase and probably only one tetramer is associated. The a-subunit would serve as an asymmetrical component to limit binding. Such interaction could account for its link to V-ATPase assembly, with aldolase triggering or stabilizing association between the a-subunit and elements of V0 and the E-subunit and B-subunit, a stator component with the membrane-distal attachment of the stator in V0.

### 3.6. Sugar Sensing by Aldolase

As described above, Aldolase binds V-ATPase and may serve to couple glycolysis to V-ATPase activity. In this section, we will describe the proposed mechanism by which aldolase is linked to energy sensing.

**Figure 6:**
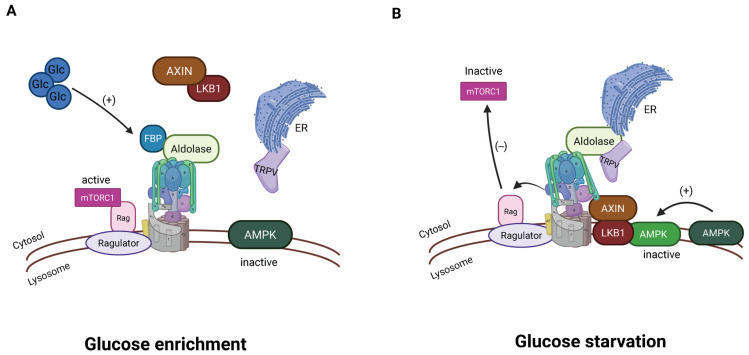
Aldolase associated with V-ATPase is involved in energy sensing. (**A**) In a high-glucose environment, V-ATPase is active, and aldolase is associated with the active V-ATPase. (**B**) When glucose is not abundant, V-ATPase is inactive; aldolase interacts with TRPV channels in the endoplasmic reticulum, inhibiting calcium release. The reduced calcium is involved in passing the signal of low glucose to the sensing system (created with BioRender.com).

Aldolase acts as a sensor for glucose availability by binding to fructose 1,6-bisphosphate (FBP), an intermediate of glucose metabolism [143,176]. Under high-glucose conditions, FBP is available for Aldolase, and this maintains the activity of the V-ATPase. While V-ATPase is active, mTORC1 is indirectly kept active as described above. When FBP is low, the Aldolase changes its conformation, and V-ATPase is no longer active [143]. V-ATPase may undergo some version of reversible assembly, as will be described below. Recently, data have emerged linking this process to calcium flux through Transient Receptor Potential V channels (TRPVs). V-ATPase-associated aldolase, when inactive due to low FBP, binds and inhibits TRPV channels in the endoplasmic reticulum [176,177] (Figure 6). This reduces local calcium and allows inactive TRPV to bind V-ATPase, which relays the FBP-free status of aldolase to the V-ATPase. In support of this mechanism, genetic depletion of TRPVs blocked glucose starvation-induced AMPK activation in cells and the liver of mice and in nematodes [177]. The pharmacological inhibitor of TRPV1–4 (AMG-9810) activated AMPK in the mice without altering the adenylate ratios and elevated NAD+ levels in aged muscles, rejuvenating the animals’ running capacity [177].

### 3.7. Role of V-ATPase Assembly and Disassembly

At the heart of current thinking regarding the role of V-ATPase in energy sensing is the hypothesis that V-ATPase activity is regulated by reversible assembly of the V-ATPase. The intact V-ATPase and disassembled subcomplexes are thought to display different binding specificities; the intact active V-ATPase stimulates mTORC1 while disassembled subcomplexes promote L-AMPK. Currently, a model system capable of studying the molecular details of this system has not yet been identified.

**Figure 7:**
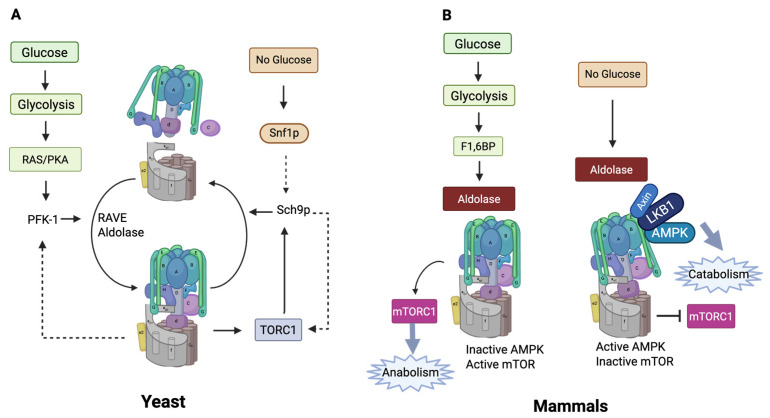
(**A**) Reversible assembly in yeast and its links to energy sensing. (**B**) Energy sensing in mammals. In mammals, V-ATPase inactivation seems to involve some form of disassembly. This is represented in the figure by a tilted V1, but the exact nature of the disassembly is not known (created with BioRender.com).

In the yeast Saccharomyces cerevisiae, V-ATPase undergoes reversible assembly that has been characterized in detail [178,179,180]. Reversible assembly of V-ATPase in yeast is linked to lysosome function [181]. The simplest model suggests that disassembly of V-ATPase involves release of the V1 subdomain into the cytosol with V0 left in the membrane. Both subdomains are locked into inactive forms; V1’s enzymatic activity is autoinhibited due to the action of the H-subunit, which prevents ATP hydrolysis [96,182]. V0 is likewise locked to prevent protons from crossing the membrane [183]. Many of the intermediate steps and the final structures have been characterized enzymatically and structurally [180,184,185,186]. In mammalian cells, characterization has been less straightforward, and though reversible assembly is proposed to regulate mammalian V-ATPases, the data are less clear [5] (Figure 7).

Consider the volume of Saccharomyces cerevisiae, 40–90 µM^3^ [187], versus a typical mammalian cell (4000 µM^3^) [188], which is 50–100 times larger. This makes clear the increased challenge in mammalian cells of reuniting a truly free V1 in the cytosol with a membrane-incorporated V0. It seems likely that scaffolding, perhaps involving microfilaments and AXIN, keeps the V-ATPase subcomplexes tethered when they are disassembled. This could conveniently provide scaffolding for the L-AMPK and LKB1. This also suggests that energy sensing may depend on the regulation of cytoskeletal dynamics. Polymerization–depolymerization and myosin-powered contraction of the cytoskeleton could have a vital role in reversible assembly. Supporting this idea, V-ATPase from osteoclasts, immunoisolated with an anti-E-subunit antibody, had both actin and myosin II bound to it [171]. Myosin II is the conventional myosin that causes contraction of the microfilament cytoskeleton.

Understanding how the activation and inactivation of V-ATPases occur in mammals is a vital challenge. Such understanding may well provide new targets for pharmacological agents to treat the myriads of pathologies associated with V-ATPase activity. Such understanding may require the identification of a model where large amounts of the energy-sensing complexes are present, or the generation of such a model to allow its isolation and structural analysis.

## 4. Energy Sensing and Periodontal Disease: How Does Inflammation Result from Energy Sensing?

### 4.1. RAGE Activation and AMPK

It is well established that increased inflammation is associated with diabetes [189]. The underlying mechanism is not clear. Several hypotheses have been presented. Hyperglycemia can stimulate inflammation [190]. High levels can cause increased levels of advanced glycation end (AGE) products on surface proteins on cells in the bloodstream [191]. The AGEs stimulate the receptor of advanced glycation end products (RAGE), which triggers oxidative stress, inflammation, and damage to organs [191]. There is a substantial body of literature that demonstrates regulatory relationships between AGE/RAGE signaling and energy sensing.

RAGE activation triggered by AGEs has been reported to downregulate AMPK activity [192]. In diabetic mice, increased AGEs and RAGE activation reduce phosphorylated AMPK. RAGE deficiency has been linked to autophagy induction through the AMPK signaling pathway [192]. AICAR, an AMPK activator, promotes the activity of the protease, ADAM10 [193]. This can lead to RAGE ectodomain shedding, inactivating the RAGE as a receptor. Likewise, metformin activates AMPK and inhibits AGE-induced RAGE activation of NF-κB [194,195].

### 4.2. Pattern Recognition Receptors (PRRs)

Innate pattern recognition receptor systems are involved in the response to periodontal infections. These include toll-like receptors (TLRs), which have been the subject of much research [196]. More recently, nucleotide-binding oligomerization domain-containing protein 1 (NOD1) was implicated in periodontal bone loss and evidence surprisingly suggested that MYD88, a molecule downstream of TLRs, was not involved [197]. Considerable evidence indicates that signaling from PRRs is entangled with energy sensing in general and with lysosomal energy sensing in particular [93,198].

There is evidence that responses triggered by TLRs are modulated by components of the lysosomal energy-sensing apparatus. For example, in response to viral double-stranded RNAs, TLR3 triggers cleavage of a regulatory portion of cellular SQSTM1/p62 [199]. SQSTM1/p62 is a ubiquitin ligase that when mutated can lead to Paget’s Disease of the bone. This cleavage is through TLR3 activation of RIPK1 and CASP8 (caspase 8). The cleaved molecule called SQSTM1/p62TRM (amino acids 1–329) is an mTORC1 regulator whose production correlates with the sustained activation of mTORC1 and RPS6KB1/p70S6K1 phosphorylation. This pathway may stimulate mTORC1 activity in excess of what is required based on nutrient status, which may lead to increased inflammatory signaling [199]. In cases where nutrient levels are already high, this enhances the anabolic signaling that leads to inflammation. However, therapeutic activation of AMPK would be expected to reduce this mTORC1 signaling by phosphorylating and activating TCS2 and phosphorylating and inhibiting RAPTOR.

NOD1 recognizes peptidoglycan fragments from bacteria in the cytosol and triggers innate immune responses [200]. The notion that a cytosolic bacterial component sensor would play a crucial role in periodontal bone loss is not surprising. One of the archetypical periodontal pathogens is *Porphyromonas gingivalis* (*P. gingivalis*), which thrives in the cytosol of periodontal cells [201].

Recently, a new mechanism has emerged that may be regulated through lysosomal energy sensing. Over time, mitochondrial damage occurs, eventually leading to leakage of double-stranded DNA from the mitochondria into the cellular cytosol. This leads to the recruitment and activation of cyclic GMP-AMP-synthase (cGAS). cGAS produces 2′3′-cGAMP (cGMP-AMP), which functions as a second messenger to activate stimulator of interferon genes (STING) [199,202]. This triggers signaling pathways resulting in the production of interferons and activation of nuclear factor kappa B (NFKB), which stimulates the production of inflammatory molecules [203] (Figure 7).

## 5. Energy Sensing and MRONJ

MRONJ is a severe pathology of the jaw that is linked primarily, but not exclusively, to agents that alter bone remodeling. The condition was first identified as bisphosphonate-related osteonecrosis of the jaw (BRONJ) that appeared in the early 2000s, caused by nitrogen-containing bisphosphonates that had recently been introduced as anti-resorptives to treat osteoporosis and bone cancer [204]. Various other agents have now been linked to the condition, including the antiresorptive, denosumab, a humanized monoclonal antibody that binds receptor activator of nuclear factor κ B (RANK) ligand and competitively inhibits its binding to its receptor RANK [205]. A second biologic, Romosozumab, which inhibits sclerostin, thereby triggering bone formation and inhibiting bone resorption, also triggers MRONJ, as does rapamycin/sirolimus [24].

For osteoporosis and bone cancer, first-line therapeutics are nitrogen-containing bisphosphonates, including zoledronate and ibandronate or denosumab [206]. Bisphosphonates function by being targeted to sites of active bone resorption, where they are incorporated into the bone [207]. When osteoclasts begin to resorb the bone, the bisphosphonate is mobilized and blocks a step in the mevalonate pathway, which prevents prenylation of Rho small GTPases and triggers apoptosis by osteoclasts [208]. Denosumab prevents osteoclast formation. The therapeutic impact of the two agents, despite their very different mechanisms of action, is similar [98,99,100]. Both also increase the risk of MRONJ about the same [24]. MRONJ occurs rarely even when these antiresorptives are given at high doses. It is hypothesized that specific genetic variations may make people susceptible, and that bisphosphonates and denosumab may have different underlying genetic susceptibilities [101]. To test this idea, studies have looked for susceptibility markers. For bisphosphonate-induced MRONJ, two genes have been identified to date. One encodes Farnesyl-Diphosphate Farnesyltransferase 1, which is an enzyme in the mevalonate pathway [102]. Altered activity of the enzyme would change the pool of substrates for the enzyme inhibited by nitrogen-containing bisphosphonates and thus alter their impact on cells. The second is Sirtuin 1 [89]. As discussed previously, this deacetylase affects lysosomal energy sensing, at least in part through inhibiting V-ATPase activity by deacetylating residues on the E-subunit [83]. The latter suggests that MRONJ susceptibility may be entangled with lysosomal energy sensing, and that agents that stimulate L-AMPK may prevent or treat MRONJ.

**Figure 8:**
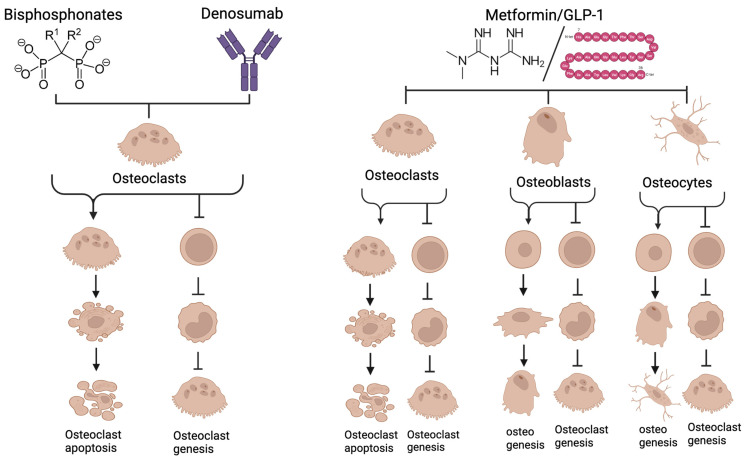
Bisphosphonates and Denosumab act directly on osteoclasts, the bone-resorbing cells. Bisphosphonates are incorporated into bone then released as osteoclasts begin resorbing. The bisphosphonate perturbs the mevalonate pathway in osteoclasts, triggering apoptosis of the osteoclast. Denosumab both blocks osteoclastogenesis and inhibits mature osteoclasts. Metformin and GLP-1 agonists have effects on all types of bone cells. They reduce differentiation and at high concentrations trigger apoptosis of osteoclasts. Both stimulate osteogenesis toward osteoblasts and osteocytes and inhibit pro-osteoclastic signals from these cells. They also affect other cells involved in periodontitis including immune cells and fibroblasts. Because of the different mechanisms of action between conventional antiresorptives and metformin/GLP-1 agonists, there may be additive or synergistic therapeutic effects. See text for more discussion (created with BioRender.com).

Bisphosphonates and denosumab inhibit bone resorption by targeting osteoclasts, while metformin and GLP-1 agonists affect not just osteoclasts but also osteoblasts, osteocytes, and all other cells in the body (Figure 8). Additive or synergistic effects could result when these different drug classes are given together. There have been several studies examining the consequences of antiresorptives in combination with metformin. Metformin and alendronate were tested in an animal model for effects on osteoporosis in a diabetic mouse model. Although both agents reduced signs of osteoporosis, there was no obvious synergy or additive effects [104]. The same combination was useful in the treatment of glucocorticoid-induced osteoporosis [103]. Promising results show that metformin given in combination with alendronate reduces cartilage damage in a mouse model of osteoarthritis [105]. Combinations of bisphosphonates with metformin have been suggested as a treatment for osteoarthritis [106]. Metformin and bisphosphonates have been tested for effects on cancer [107,108]. A practical effect of metformin is that it reduces gastric damage associated with oral bisphosphonates in rat models [109]. Surprisingly, there is very limited data regarding the use of the combination of bisphosphonates with denosumab/GLP-1 agonists with antiresorptives [209]. Interestingly, denosumab alone increases intrinsic GLP-1 levels and improves glucose control in postmenopausal women with type 2 diabetes [210].

There is some evidence supporting the use of metformin for treating MRONJ [40,41]. There is also ample evidence that diabetes is a risk factor for MRONJ [110,142,207]. Based on this evidence, further study of agents or practices that encourage L-AMPK activation for preventing and treating MRONJ is warranted.

It must be noted, however, that rapamycin has been implicated in the development of MRONJ, although with low risk [24]. More detailed understanding of lysosomal energy sensing and how it interacts with periodontitis and other diseases is urgently needed. Lysosomal energy sensing is of great importance to the cell and to the mammalian organism. The machinery is complex and in many areas is not understood [4]. The complexity of the signaling may provide targets for future therapeutic agents or therapeutic approaches.

## 6. Conclusions

Remarkable progress in understanding how cells respond to nutrients and how this impacts the health of the body in general and periodontal health has been accomplished. The nutrient sensing machinery is very ancient. In budding yeast and plants, it is significantly different from that in mammalian cells, but V-ATPase has served as the core of the machinery since the rise of eukaryotic cells. Shifting cellular responses to varying nutrient availability allows cells to adapt to the environment. In humans, an overabundance of nutrients can lead to increased inflammation. Activation of cGAS/STING downstream of mitochondrial damage and insufficient mitophagy were recently described and add to oxidative stress and AGE/RAGE signaling associated with high levels of carbohydrates.

Diabetes and obesity were identified as risk factors for periodontal disease before the dawn of molecular cell biology. Lysosomal energy sensing provides a mechanistic basis to at least partially explain this link.

Much is unknown regarding lysosomal energy sensing, including fundamentally important elements. Perhaps most important is the fact that reversible assembly, or whatever changes in V-ATPase that occur in mammals to regulate its activity, is not well understood. It is also not known whether and how V-ATPase isoforms influence association with nutrient-sensing machinery. The interactions between V-ATPase and the energy-sensing complexes that signal high and low energy states are not yet fully understood. Indeed, a central focus appears to be *ATP6AP1*. This subunit was recently considered to be an accessory protein to V-ATPase but now appears to be a core subunit.

Therapeutics, including metformin and GLP-1 agonists, show promise for the prevention and treatment of periodontal disease and MRONJ, particularly in diabetic patients. However, these medications have been associated with off-target effects. For example, GLP-1 agonists have been linked to gastrointestinal issues, pancreatitis, increased risk of depression and suicide, nephrolithiasis or kidney disease, and, in a few cases, acute kidney disease downstream of dehydration from gastrointestinal issues [113,114]. The effectiveness of metformin and GLP-1 agonists serves as a mechanistic basis for clinicians to encourage patients to engage in activities that promote a healthy blend of L-AMPK and mTORC1 activity. In many, this can be obtained and maintained by modest increases in exercise and improvements in diet [211].

While we typically think of advancing medicine as developing new therapeutic agents, lysosomal energy-sensing pathways and their role in health offer an example where behavioral changes obviously offer opportunities to prevent or treat periodontal disease and possibly MRONJ. For example, the underlying mechanistic basis for increased health and longevity observed on a calorically restricted diet was recently uncovered, and it involved a pathway to inhibit V-ATPase and thereby activate L-AMPK [83]. While it is possible that lithocholic acid, the agonist for sirtuins that was induced by caloric restriction, might be developed into a therapeutic agent, caloric restriction might be a simpler and safer solution. Treatment of periodontitis and MRONJ might be easily and naturally accomplished by eating healthier, staying away from sweets, reducing caloric intake, getting plenty of exercise, and perhaps practicing intermittent fasting [212]. Because of the complexity of the lysosomal energy-sensing machinery, the safest and most efficient approach may be finding ways to adjust the natural energy rheostat through changes in behavior.

## Figures and Tables

**Table 1:** Conservation of elements of lysosomal energy-sensing apparatus in eukaryotes.

Elements of V1 subdomain of V-ATPase										
	*ATP6V1A*	*ATP6V1B2*	*ATP6V1C1*	*ATP6V1D*	*ATP6V1E1*	*ATP6V1F*	*ATP6V1G1*	*ATP6V1H*		
Homo sapiens (mammal)	100	100	100	100	100	100	100	100		
Mus musculis (mammal)	98	100	100	98	99	98	95	99		
Drosphila melanogaster (insect)	83	91	65	72	63	72	50	53		
Arabidopsis thaliana (land plant)	69	91	40	54	41	50	39	28		
Saccharomyces cerevisiae (budding yeast)	67	81	39	51	34	53	38	31		
Elements of V0 subdomain of V-ATPase										
	*ATP6V0a1*	*ATP6V0a2*	*ATP6V0a3*	*ATP6V0a4*	*ATP6V0b*	*ATP6V0c*	*ATP6v0d1*	*ATP6V0e1*	*ATP6AP1*	*ATP6AP2*
Homo sapiens (mammal)	100	100/57	100/53	100/61	100	100	100	100	100	100
Mus musculis (mammal)	96/a1	92/a2	84/a3	86/a4	99	91	100	100	86	94
Drosphila melanogaster (insect)	62/a1	45/a2	43/a1	54/a1	64	78	81	41	29	26
Arabidopsis thaliana (land plant)	43/a3	38/a1	41/a3	42/a2	56	62	51	46		
Saccharomyces cerevisiae (budding yeast)	42/*vph1*	37/*vph1*	36/*vph1*	38/*vph1*	34	63	46	46		
Elements of mTORC1 and L-AMPK										
	RAPTOR	Tor	LST8	SIN1	RICTOR	Rheb	TSC	AMPK-α	AMPK-β	AMPK-γ
Homo sapiens (mammal)	100	100	100	100	100	100	100	100	100	100
Mus musculis (mammal)	93	99	98	97	95	99	87	99	97	97
Drosphila melanogaster (insect)	32	54	49	29	32	64	32	46	62	66
Arabidopsis thaliana (land plant)	41	42	45					53	38	30
Saccharomyces cerevisiae (budding yeast)	25	44/42	47	32	25	37		41	31	37

Percent identity compared with the canonical sequence of the human protein from UniProt using BLAST (National Library of Medicine), with two exceptions. First, human *ATP6V0a* subunits are also compared with human *ATP6V0a1* to show the conservation of human a-subunit isoforms. Second, the two isoforms of yeast Tor are compared with human Tor.

## Data Availability

No new data were created or analyzed in this study.

## Abbreviations

The following abbreviations are used in this manuscript:

V-ATPase	vacuolar H+-ATPase
L-AMPK	lysosomal AMP-activated protein kinase complex
mTORC1	mammalian target of rapamycin complex 1
MRONJ	medicine-related osteonecrosis of the jaw
RAG	Ras-related GTPase
AXIN	axis inhibition protein
GLP-1	Glucagon-like Peptide-1
FOXO	Forkhead Box O
2-DG	2-Deoxy-d-glucose
TDLc2	TBC/LysM-Associated Domain Containing 2
mEAK7	Mammalian Enhancer-of-Akt-1–7
RAPTOR	regulatory-associated protein of mTOR
MLST8	mammalian lethal with sec-13 protein 8
PRAS40	proline-rich Akt substrate of 40 kDa
TTI1	Tel2 interacting protein 1
TEL2	Telomere maintenance 2 interacting protein 2
DEPTOR DEP	domain containing mTOR-interacting protein
RICTOR	Rapamycin-insensitive companion of mTOR
PROCTOR	protein observed with RICTOR
LKB-1	Liver Kinase B1
PEN2	presenilin enhancer 2
TSC2	Tuberous sclerosis complex 2
GEF	Guanine exchange factor
GAP	GTPase-activating protein
SLC38A9	Solute carrier family 38 member 9
GATOR	GTPase-activating protein toward Rags
Rheb	Ras homolog enriched in brain
PI-3 kinase	Phosphatidylinositol 3-kinase
AKT	Protein kinase B
LAMTOR	Late endosomal/lysosomal adaptor and MAPK and mTOR activator
TRPV	Transient Receptor Potential V
AGE	Advanced glycation end product
PRR	Pattern Recognition Receptors
TLR	Toll-like receptor
NOD1	nucleotide-binding oligomerization domain-containing protein 1
cGAS	cyclic GMP-AMP-synthase
STING	Stimulator of Interferon Genes
BRONJ	bisphosphonate-related osteonecrosis of the jaw
RANK	receptor activator of nuclear factor κ B
